# Antigenic Variation of Avian Influenza A(H5N6) Viruses, Guangdong Province, China, 2014–2018

**DOI:** 10.3201/2510.190274

**Published:** 2019-10

**Authors:** Ru Bai, Reina S. Sikkema, Cong rong Li, Bas B. Oude Munnink, Jie Wu, Lirong Zou, Yi Jing, Jing Lu, Runyu Yuan, Ming Liao, Marion P.G. Koopmans, Chang-wen Ke

**Affiliations:** Guangdong Provincial Center for Disease Control and Prevention, Guangzhou, China (R. Bai, C. Li, J. Wu, L. Zou, Y. Jing, J. Lu, R. Yuan, C. Ke);; Erasmus Medical Centre, Rotterdam, the Netherlands (R.S. Sikkema, B.B. Oude Munnink, M.P.G. Koopmans);; Southern Medical University Guangzhou (C. Li, Y. Jing, C. Ke);; South China Agricultural University, Guangzhou (M. Liao)

**Keywords:** Antigenic variation, Guangdong Province, H5N6, avian influenza, China, zoonoses, viruses, poultry, live poultry markets, chickens

## Abstract

Market surveillance showed continuing circulation of avian influenza A(H5N6) virus in live poultry markets in Guangdong Province in 2017, despite compulsory vaccination for avian influenza A(H5Nx) and A(H7N9). We analyzed H5N6 viruses from 2014–2018 from Guangdong Province, revealing antigenic drift and decreased antibody response against the vaccine strain in vaccinated chickens.

Human disease from low-pathogenic influenza A(H7N9) infection was first reported in 2013, and a total of 1,567 human cases have been reported (*1*). During the fifth wave, which started in October 2016, the number of human cases increased steeply, the virus spread into western provinces of China, and a highly pathogenic avian influenza (HPAI) A(H7N9) variant emerged (*2*). In parallel, HPAI H5 subtype viruses (clade 2.3.4.4 H5Nx) were causing international outbreaks in poultry (*3**,**4*) and infecting humans in China (*5**,**6*). In July 2017, Guangdong Province implemented a compulsory vaccination strategy for poultry (chickens, ducks, geese, quail, pigeons, and rare birds in captivity) using the combined inactivated influenza vaccine (H5 A/chicken/Guizhou/4/2013 [Re-8] + H7 A/Pigeon/Shanghai/S1069/2013 [Re-1]) to prevent the dissemination of HPAI A(H7N9) and A(H5Nx) viruses (*7*).

Our market surveillance showed that H7N9 viruses almost disappeared from live poultry markets (LPMs), although low-level circulation in poultry and the environment, as well as sporadic human cases, are still reported throughout China (*8**,**9*). However, during the same period, H5N6 subtype viruses continued to circulate in LPMs. We report our investigation of the prevalence, evolution, and antigenic variation of H5N6 viruses during 2014–2018 in Guangdong Province.

## The Study

**Figure 1:**
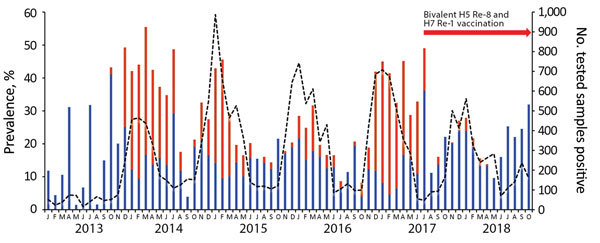
Proportion of H5 (blue bars) and H7 (red bars) subtypes in avian influenza A virus–positive samples (dashed line) from live poultry markets, Guangdong province, China, January 2013–October 2018. Re-8, A/chicken/Guizhou/4/2013 (Re-8); Re-1, H7 A/pigeon/Shanghai/S1069/2013 (Re-1).

To investigate the emergence and spread of HPAI H7N9 and H5Nx viruses in LPMs, we collected environmental and poultry samples and a throat swab from an H5N6-infected person in September 2018. We tested samples using reverse transcription PCR (RT-PCR) and real-time RT-PCR (rRT-PCR) to distinguish between subtypes H5 and H7. During January 2016–October 2018, a total of 52,387 environmental samples were collected, of which 1,627 (3.1%) were positive for H5 and 1,303 (2.5%) for H7. All H7-positive samples were of the H7N9 subtype, and 99% of H5-positive samples were of the H5N6 subtype (Figure 1; Appendix 1 Table 1). After implementation of poultry vaccination the rate of H7N9 virus–positive samples decreased from 12.8% to 0%, and the average positivity rate for H5-subtype viruses remained ≈20% (Figure 1; Appendix 1 Table 1).

We cultured 883 H5 subtype–positive samples, including the human H5N6 sample. Virus cultivation was successful for 147 environmental samples, 21 poultry samples, and the human sample. We selected 73 H5N6 isolates that were amplified successfully for whole-genome sequencing using the Ion PGM system and the PathAmp FluA reagents (Life Technologies, https://www.thermofisher.com). We analyzed data using CLC Genomics Workbench 7.5.1 software (QIAGEN, https://www.qiagenbioinformatics.com). 

**Figure 2:**
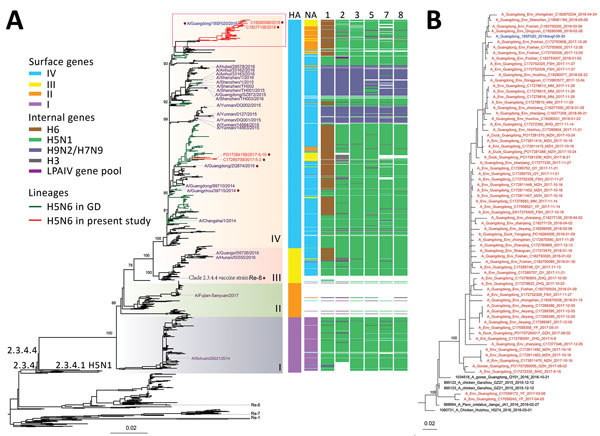
Phylogeny of influenza A(H5N6) viruses collected in Guangdong Province, China, January 2013–October 2018, compared with reference isolates. A) Viruses of clade 2.3.4.4 H5N6 viruses are divided into 4 subgroups (I–V) on the basis of the surface genes (HA and NA). Colors in key distinguish surface and internal genes. The A/chicken/Guizhou/4/2013 (Re-8) vaccine strain and viral strains used for HI testing are labeled. The 2018 human H5N6 isolate from Guangdong Province is blue, human H5N6 virus sequences since 2013 are purple, human and environmental H5N6 isolates used for the HI test are labeled with a purple dot (except for HA256 human strain, for which no sequence was available). The top part of the tree containing the bulk of the Guangdong Province recent H5N6 viruses and the human case is highlighted with a red box. All branch lengths are scaled according to the number of substitutions per site. Scale bar indicates nucleotide substitutions per site. GD, Guangdong; HA, hemagglutinin gene; LPAIV, low pathogenicity avian influenza virus; NA, neuraminidase gene; 1, polymerase basic 2 gene; 2, polymerase basic 1 gene; 3, polymerase acidic gene; 5, nucleoprotein gene; 7, matrix gene; 8, nonstructural gene. B) An expansion of the phylogenetic tree in the red outlined box of panel A. The sequence in blue is the newly approved vaccine strain 18SF020.

We combined genome sequences from this study with all sequences of H5N6 viruses from China, as well as H3 and H6 subtype viruses available in GenBank and the GISAID database (https://www.gisaid.org) for 1996–2018 (Appendix 2 Tables 1, 2). For sequencing, we used MUSCLE version 3.5 (*10*) and phylogenetic analysis under the general time reversible plus invariant sites plus Γ4 model (hemagglutinin [HA], neuraminidase [NA], polymerase basic [PB] 1, PB2, polymerase acidic [PA], nucleoprotein [NP]) and the transversion model plus F plus invariant sites plus Γ4 model (matrix [M], nonstructural [NS]), performed using IQ-TREE (*11*). Phylogenetic analysis showed that all H5N6 viruses isolated in Guangdong Province descended from the H5N6 viruses that circulated in the province during 2015–2016. However, the currently circulating H5N6 viruses in Guangdong Province cluster separately from the A/chicken/Guizhou/4/2013 (Re-8) vaccine strain, based on HA sequences (Figure 2). All N6 genes belong to the Eurasian lineage. Both HA and NA genes of the human H5N6 virus clustered with the H5N6 viruses found in the environment and poultry in our study (Appendix 1 Figure).

We classified both surface and internal genes of HPAI H5N6 viruses from Guangdong Province into different sublineages according to tree topology and bootstrap values of >85% and further classified the HA and NA genes into 4 subgroups (Figure 2). Phylogeny of the internal genes of the recent clade 2.3.4.4 H5N6 viruses showed they evolved from H5N1 viruses from 2013–2014, in which, from 2015 onward, almost all PB2 genes were replaced by H6 subtype–origin PB2 genes. Substitution of the PB2 gene can change the virulence and pathogenicity in mammals and in different bird species (*12*). Moreover, from 2016 onward, H5N6 acquired PB1 and PA genes from (avian) H3-like or LPAI gene pools. In 2017, NP, M, and NS genes from H3-like viruses and local LPAI gene pools were first detected in circulating H5N6 viruses (Figure 2). Closely related H5N6 viruses from China with similar internal gene composition did not show any intravenous pathogenicity in ducks and lower intravenous pathogenicity in chickens (*13*), which could explain the widespread circulation of H5N6 viruses in Guangdong Province.

When we compared the HA gene predicted receptor binding sites and other regions of the H5N6 isolates from Guangdong Province with A/chicken/Guizhou/4/2013 (Re-8), we found 35 positions where >50% of viruses in our study had amino acid substitutions (*4*). Those mutations (H3 numbering) occurred in sites R50K, D63N, R81S, S94A/T, L122Q, S125R/K, P128S, D129N/S, D130 deletion/E/T, T131S, L133 deletion/S, A137T, A138S, Q142K, M144V, P145A, I155T, N158S, T160A, R173G/K, S185P, N187S, A188V, A189E, T192A, N193D/K/T/N, T199A/I, R227S/C/Q/G, K238R, V260I, K262T, M272I, H276K/N/Q/S, N278S, and N323S (Appendix 1 Table 2). In addition, we detected several mutations that were exclusively found in >90% of the most recent H5N6 isolates from Guangdong Province (2017–2018), including L122Q, S125R/K, P128S, P145A, K262T, M272I, H276K/N/Q/S, and N401I/S/N. We identified 3 new amino acid substitutions in the NA and PB2 genes of human H5N6 isolate: the Q136H on the NA gene, which might affect its susceptibility to antiviral neuraminidase inhibitors (*4*), and mutation E627V and A588V in the PB2 gene, of which the influence on its virulence in mammals needs further investigation (*4*). Furthermore, we found A588V mutations in 64 of 68 PB2 genes of H5N6 viruses from the environment.

We determined HI titers in serum of H5 A/chicken/Guizhou/4/2013 (Re-8)–vaccinated chickens (n = 5) and serum from the H5N6 virus–infected human patient to human and environmental H5N6 viral isolates from different time points using a standard protocol (*14*). Serum from chickens vaccinated with H5 A/chicken/Guizhou/4/2013 (Re-8) showed high titers (8–10 log_2_) to the human H5N6 isolates from 2014–2017 and lower titers (4–6 log_2_) to the human H5N6 isolate from 2018. We observed a similar trend when using environmental isolates for the HI assays. Conversely, serum from the H5N6-infected human in 2018 showed higher titers to human H5N6 isolates in 2017 and 2018 (6 log_2_) than to those from 2014 and 2015 (4 log_2_) (Table).

**Table:** HI titers of influenza A(H5N6) virus strains collected during 2014–2018 in Guangdong Province, China, compared with vaccine strains*

Virus strain†	Sample type	Collection date	HI titers						
			Postvaccination chicken serum						H5N6-infected human serum
			S1	S2	S3	S4	S5		S6
39715	Human	2014 Dec 11	512	512	1024	512	256		16
ZQ874	Human	2015 Dec 31	512	512	512	512	256		16
HA256	Human	2017 Jun 30‡	256	256	512	256	256		64
18SF020-1	Human	2018 Sep 30	32	16	64	32	16		64
C17280709	Environment	2017 May 2	64	64	128	128	64		16
C18277136	Environment	2018 Apr 2	64	32	32	64	16		16
C18285099	Environment	2018 Feb 28	32	32	NT	NT	32		8
PO17284158	Waterfowl	2018 May 10	128	64	NT	NT	NT		NT
A§	Chicken	2018 Nov 19¶	2,048	1,024	4,096	1,024	1,024		NT
B#	Chicken	2018 Sep 20**	32	32	NT	NT	NT		NT

*NT, not tested; S, sample no. †The name of virus strain is the abbreviation of the original name for each viral isolate. ‡Date isolate received. §Vaccine strain A/chicken/Guizhou/4/2013 (Re-8) + H7 A/pigeon/Shanghai/S1069/2013 (Re-1). ¶Date vaccine strain tested. #Vaccine strain H5 A/duck/Guangdong/S1322/2010 (Re-6). **Date vaccine strain received.

## Conclusions

Compulsory vaccination of the combined inactivated influenza vaccine was implemented in Guangdong Province in July 2017. Although the prevalence of H7N9 in LPMs decreased abruptly, we revealed uninterrupted circulation of H5N6 viruses in LPMs after implementation of the vaccination strategy. Our study shows that H5N6 viruses in Guangdong Province show antigenic drift when compared with the A/chicken/Guizhou/4/2013 (Re-8) vaccine strain, resulting in lower protection of vaccinated chickens against circulating clade 2.3.4.4 H5 viruses. In December 2018, the China government approved a new poultry vaccine (H5 A/duck/Guizhou/S4184/2017 [Re-11], H5 A/chicken/Liaoning/SD007/2017 [Re-12] + H7 A/chicken/Guangxi/SD098/2017 [Re-2]). Moreover, the World Health Organization proposed a new A/Guangdong/18SF020/2018-like H5N6 candidate vaccine virus, which was partly based on strain A/Guangdong/18SF020/2018 reported in this study (*15*).

Vaccine escape variants remain a risk for human and animal health. Therefore, future policy should focus on preventing the spread of avian influenza viruses along the market chain by strengthening farm-level surveillance and biosecurity, as well as implementing measures to monitor and prevent the spread of avian influenza viruses that have zoonotic potential in the market chain.

Appendix 1 Additional methods and results for study of antigenic variation of avian influenza A(H5N6) viruses, Guangdong Province, China, 2014–2018.

Appendix 2 Genomes from this study and from the GenBank and GISAID databases.
