# Supplementary material for: Antigenic Variation of Avian Influenza A(H5N6) Viruses, Guangdong Province, China, 2014–2018
Source: Emerg Infect Dis. 2019 Oct;25(10):1932–45. doi: 10.3201/2510.190274 (PMC6759240; doi:10.3201/2510.190274)
Supplement: Appendix 1 — Additional methods and results for study of antigenic variation of avian influenza A(H5N6) viruses, Guangdong Province, China, 2014–2018. [file 19-0274-Techapp-s1.pdf]

Article DOI: https://doi.org/10.3201/eid2510.190274

Antigenic Variation of Avian Influenza A(H5N6) Viruses, Guangdong Province, China, 2014–2018

Appendix 1

Appendix 1 Table 1. Epidemiologic surveillance of H5, H7, and avian influenza A viruses, Guangdong province, China, January 2013–October 2018*

Time	No. tested samples	FluA+, no. (%)	H5/FluA+, no. (%)	H7/FluA+, no. (%)
Jan 2013	108	51 (47.22)	6 (11.76)	0 (0)
Feb 2013	81	23 (28.4)	1 (4.35)	0 (0)
Mar 2013	126	38 (30.16)	4 (10.53)	0 (0)
Apr 2013	230	74 (32.17)	23 (31.08)	0 (0)
May 2013	339	72 (21.24)	1 (1.39)	0 (0)
June 2013	182	15 (8.24)	1 (6.67)	0 (0)
July 2013	184	41 (22.28)	13 (31.71)	0 (0)
Aug 2013	256	66 (25.78)	1 (1.52)	0 (0)
Sept 2013	194	47 (24.23)	7 (14.89)	0 (0)
Oct 2013	196	51 (26.02)	21 (41.18)	1 (1.96)
Nov 2013	261	75 (28.74)	15 (20)	0 (0)
Dec 2013	656	242 (36.89)	61 (25.21)	58 (23.97)
Jan 2014	1,978	457 (23.1)	56 (12.25)	136 (29.76)
Feb 2014	1,800	464 (25.78)	44 (9.48)	160 (34.48)
Mar 2014	1,499	437 (29.15)	73 (16.7)	169 (38.67)
Apr 2014	1,436	328 (22.84)	45 (13.72)	94 (28.66)
May 2014	852	172 (20.19)	27 (15.7)	37 (21.51)
June 2014	489	147 (30.06)	20 (13.61)	31 (21.09)
July 2014	403	109 (27.05)	32 (29.36)	21 (19.27)
Aug 2014	403	126 (31.27)	15 (11.9)	7 (5.56)
Sept 2014	386	155 (40.16)	6 (3.87)	0 (0)
Oct 2014	401	151 (37.66)	29 (19.21)	3 (1.99)
Nov 2014	953	336 (35.26)	70 (20.83)	39 (11.61)
Dec 2014	1,377	548 (39.8)	91 (16.61)	59 (10.77)
Jan 2015	3,776	987 (26.14)	140 (14.18)	283 (28.67)
Feb 2015	3,279	655 (19.98)	63 (9.62)	235 (35.88)
Mar 2015	3,206	464 (14.47)	47 (10.13)	88 (18.97)
Apr 2015	2,689	526 (19.56)	76 (14.45)	27 (5.13)
May 2015	2,236	365 (16.32)	37 (10.14)	23 (6.3)
June 2015	934	139 (14.88)	22 (15.83)	6 (4.32)
July 2015	623	117 (18.78)	18 (15.38)	0 (0)
Aug 2015	758	119 (15.7)	17 (14.29)	2 (1.68)
Sept 2015	617	105 (17.02)	13 (12.38)	2 (1.9)
Oct 2015	636	121 (19.03)	26 (21.49)	0 (0)
Nov 2015	1,407	361 (25.66)	62 (17.17)	2 (0.55)
Dec 2015	2,080	644 (30.96)	122 (18.94)	9 (1.4)
Jan 2016	2,523	743 (29.45)	161 (21.67)	50 (6.73)
Feb 2016	2,186	537 (24.57)	81 (15.08)	52 (9.68)
Mar 2016	2,203	611 (27.73)	109 (17.84)	84 (13.75)
Apr 2016	1,755	342 (19.49)	55 (16.08)	12 (3.51)
May 2016	1,867	430 (23.03)	54 (12.56)	17 (3.95)
June 2016	625	85 (13.6)	11 (12.94)	3 (3.53)
July 2016	596	105 (17.62)	7 (6.67)	2 (1.9)
Aug 2016	750	132 (17.6)	15 (11.36)	0 (0)
Sept 2016	572	97 (16.96)	19 (19.59)	1 (1.03)
Oct 2016	712	99 (13.9)	4 (4.04)	4 (4.04)
Nov 2016	1,852	421 (22.73)	52 (12.35)	27 (6.41)
Dec 2016	2,590	686 (26.49)	81 (11.81)	206 (30.03)
Jan 2017	3,790	708 (18.68)	58 (8.19)	260 (36.72)
Feb 2017	3,341	672 (20.11)	31 (4.61)	247 (36.76)

Time	No. tested samples	FluA+, no. (%)	H5/FluA+, no. (%)	H7/FluA+, no. (%)
Mar 2017	3,062	507 (16.56)	33 (6.51)	131 (25.84)
Apr 2017	1,910	355 (18.59)	59 (16.62)	101 (28.45)
May 2017	1,960	286 (14.59)	44 (15.38)	38 (13.29)
June 2017	599	55 (9.18)	6 (10.91)	12 (21.82)
July 2017	632	47 (7.44)	17 (36.17)	6 (12.77)
Aug 2017	581	90 (15.49)	10 (11.11)	0 (0)
Sept 2017	631	94 (14.9)	13 (13.83)	2 (2.13)
Oct 2017	809	168 (20.77)	37 (22.02)	0 (0)
Nov 2017	1,831	501 (27.36)	101 (20.16)	1 (0.2)
Dec 2017	1,758	412 (23.44)	100 (24.27)	11 (2.67)
Jan 2018	2,164	561 (25.92)	134 (23.89)	22 (3.92)
Feb 2018	1,892	324 (17.12)	62 (19.14)	8 (2.47)
Mar 2018	1,751	239 (13.65)	32(13.39)	5 (2.09)
Apr 2018	1,776	262 (14.75)	35 (13.36)	1 (0.38)
May 2018	1,929	283 (14.67)	27 (9.54)	0 (0)
June 2018	655	69 (10.53)	11 (15.94)	0 (0)
July 2018	705	111 (15.74)	28 (25.23)	0 (0)
Aug 2018	656	141 (21.49)	31 (21.99)	0 (0)
Sept 2018	791	237 (29.96)	58 (24.47)	0 (0)
Oct 2018	933	160 (17.15)	51 (31.88)	0 (0)
Total	8,9418	19,398 (15.73)	2,932 (11.08)	2,795 (6.28)

*Flu A+, type A avian influenza viruses; H5, H5 subtype avian influenza virus; H7, H7 subtype avian influenza virus.

Appendix 1 Table 2. Amino acid substitution of the hemagglutinin protein of influenza A(5N6) viruses from human and environment compared with vaccine strain A/Chicken/Guizhou/4/2013 (Re-8) (H3 numbering)

	Amino acids of viruses (no.)		
Molecular feature or amino acid substitution	Re-8 (1)	Human (1)	Environment (72)
Multibasic cleavage site			
PLRERRRKR↓G			
Amino acid substitution			
R50K	R	K	K (67), R(5)
D63N	D	N	D (47), N (25)
R81S	R	S	R (26), S (46)
S94A/T	S	A	A (68), T (4)
L122Q	L	Q	L (7), Q (65)
S125R/K	S	R	R (57), K (2), S (13)
P128S	P	S	S (59), P (13)
D129N/S	D	N	N (69), S (3)
D130bdeletion/E/T	D	-	65 (–), E (5), T (2)
T131S	T	T	T (70), S (2)
L133 deletion/S	L	S	7 (–), S (65)
A137T	A	A	T (1), A (71)
A138S	A	A	S (1), A (71)
Q142K	Q	Q	Q (67), K (5)
M144V	M	V	V (70), M (2)
P145A	P	A	A (59), P (13)
I155T	I	T	T (72)
N158S	N	N	S (1), N (71)
T160A	T	A	A (72)
R173G/K	R	K	G (5), K (56),
S185P	S	S	P (3), S (69)
N187S	N	S	S (60), N (12)
A188V	A	A	V (1), A (71)
A189E	A	E	E (71), A (1)
T192A	T	T	A (8), T (64)
N193D/K/T/N	N	D	D (26), K (10), T (1), N (35)
T199A/I	T	T	T (70), I (1), A (1)
R227S/C/Q/G	R	R	S (4), C (2), Q (5), R (58), G (3)
K238R	K	R	R (62), K (10)
V260I	V	I	I (67), V (5)
K262T	K	T	T (67), K (5)
M272I	M	I	I (63), M (9)
H276K/N/Q/S	H	N	K (2), N (60), Q (1), S (5), H (4)
N278S	N	N	N (71), S (1)
N323S	N	S	S (72)

*–, deletion.

A

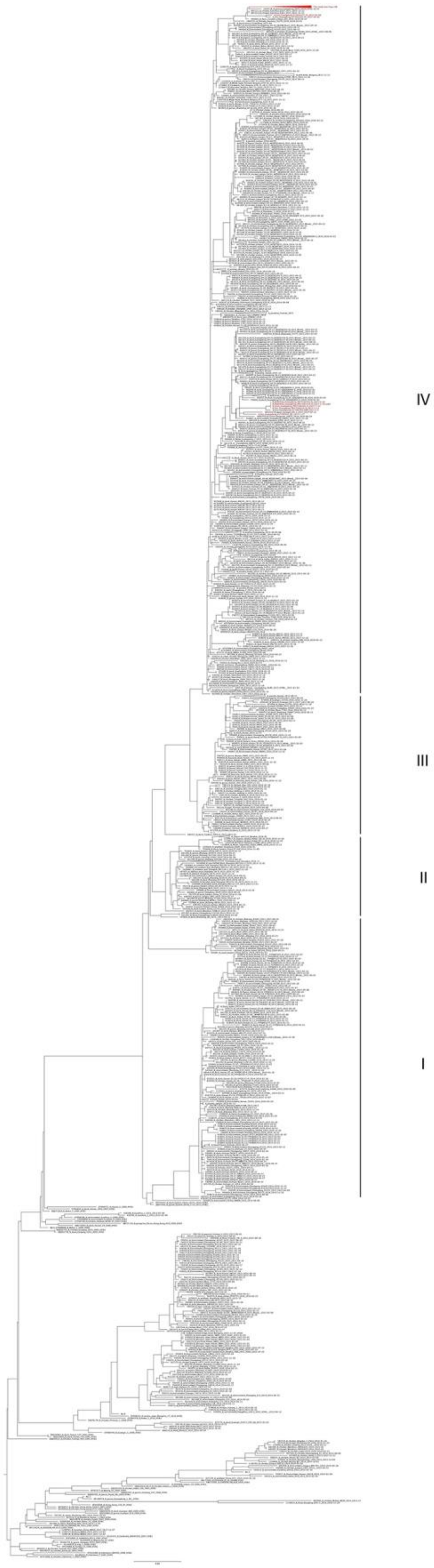

B

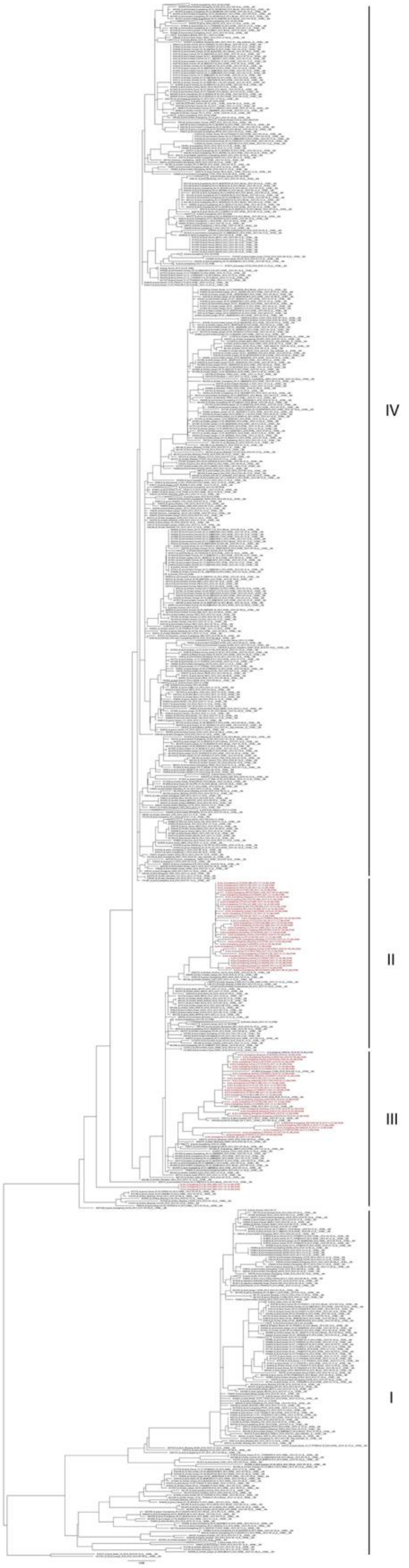

C

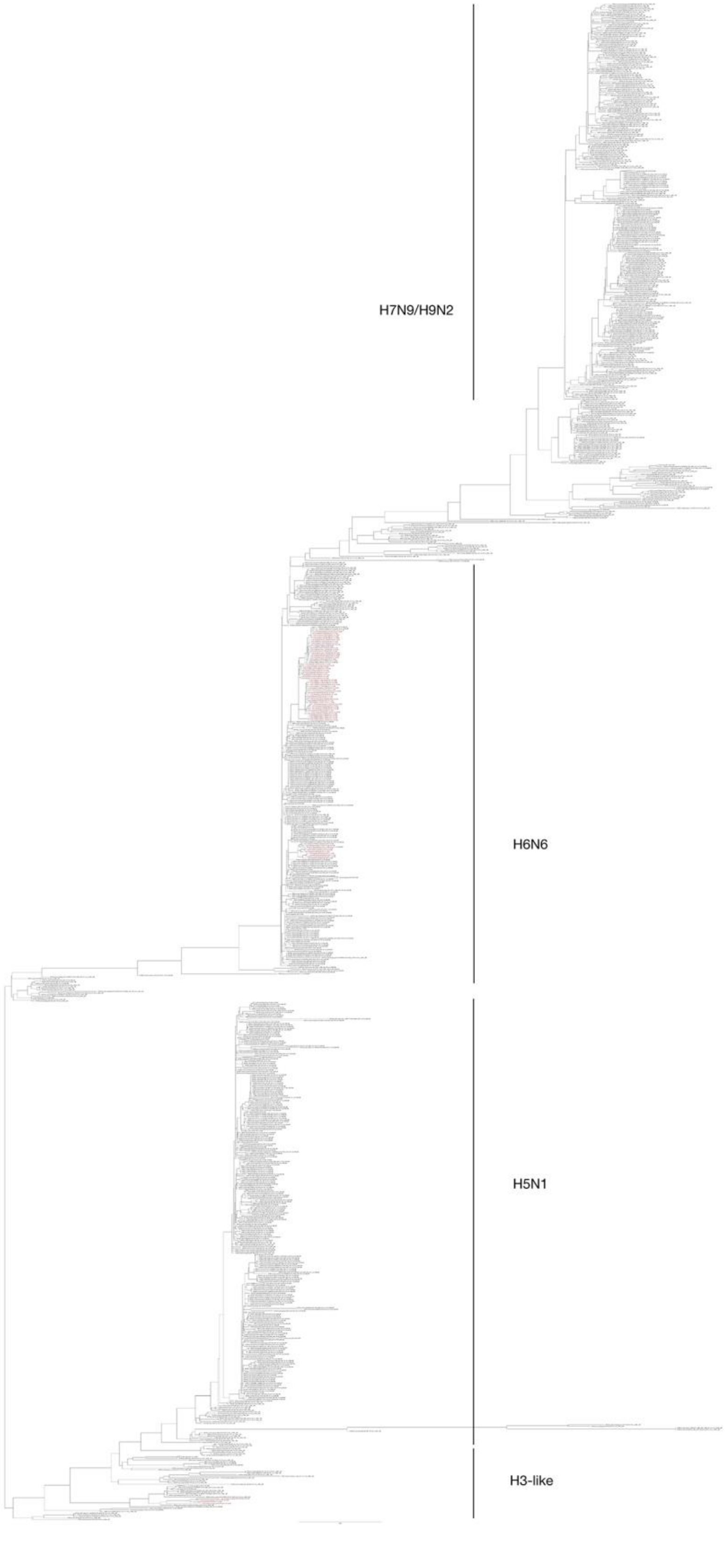

D

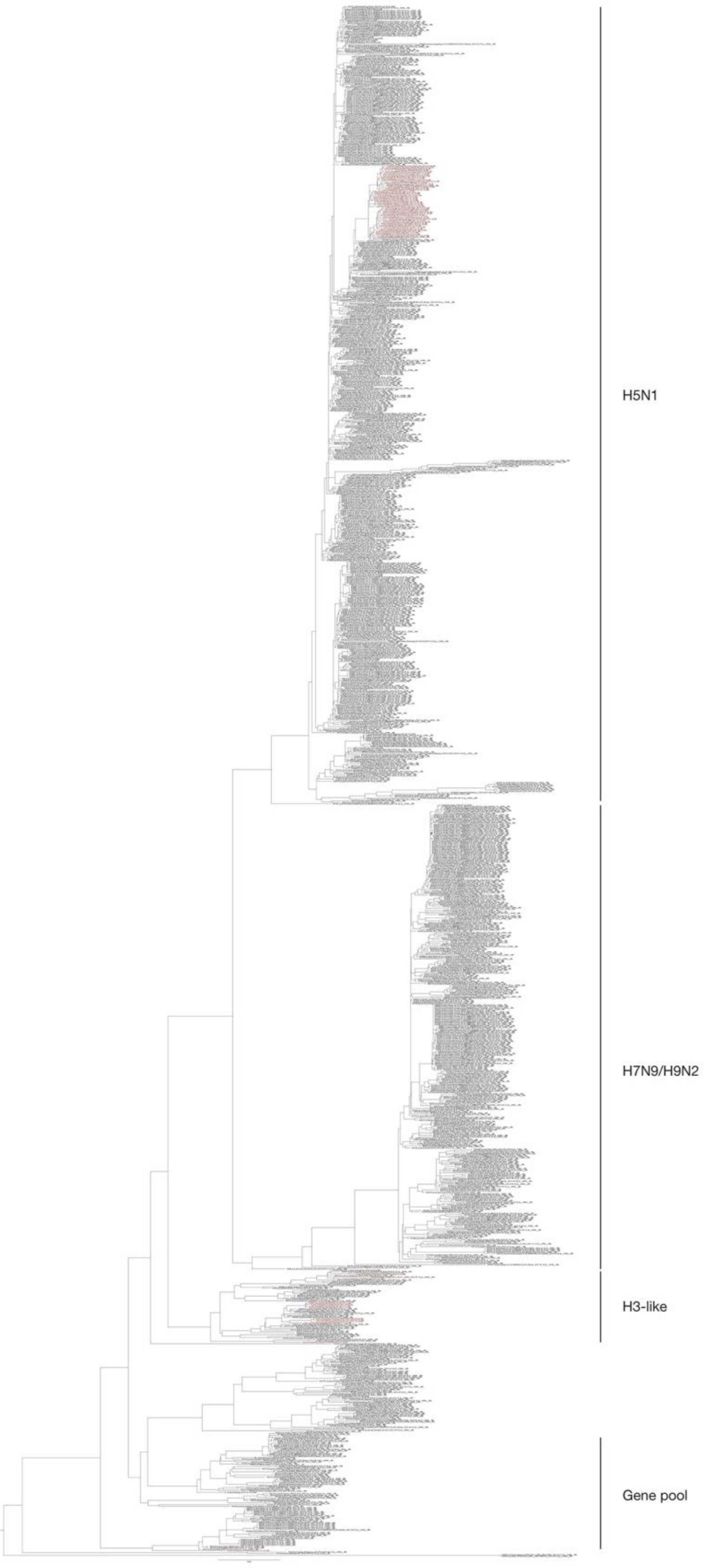

E

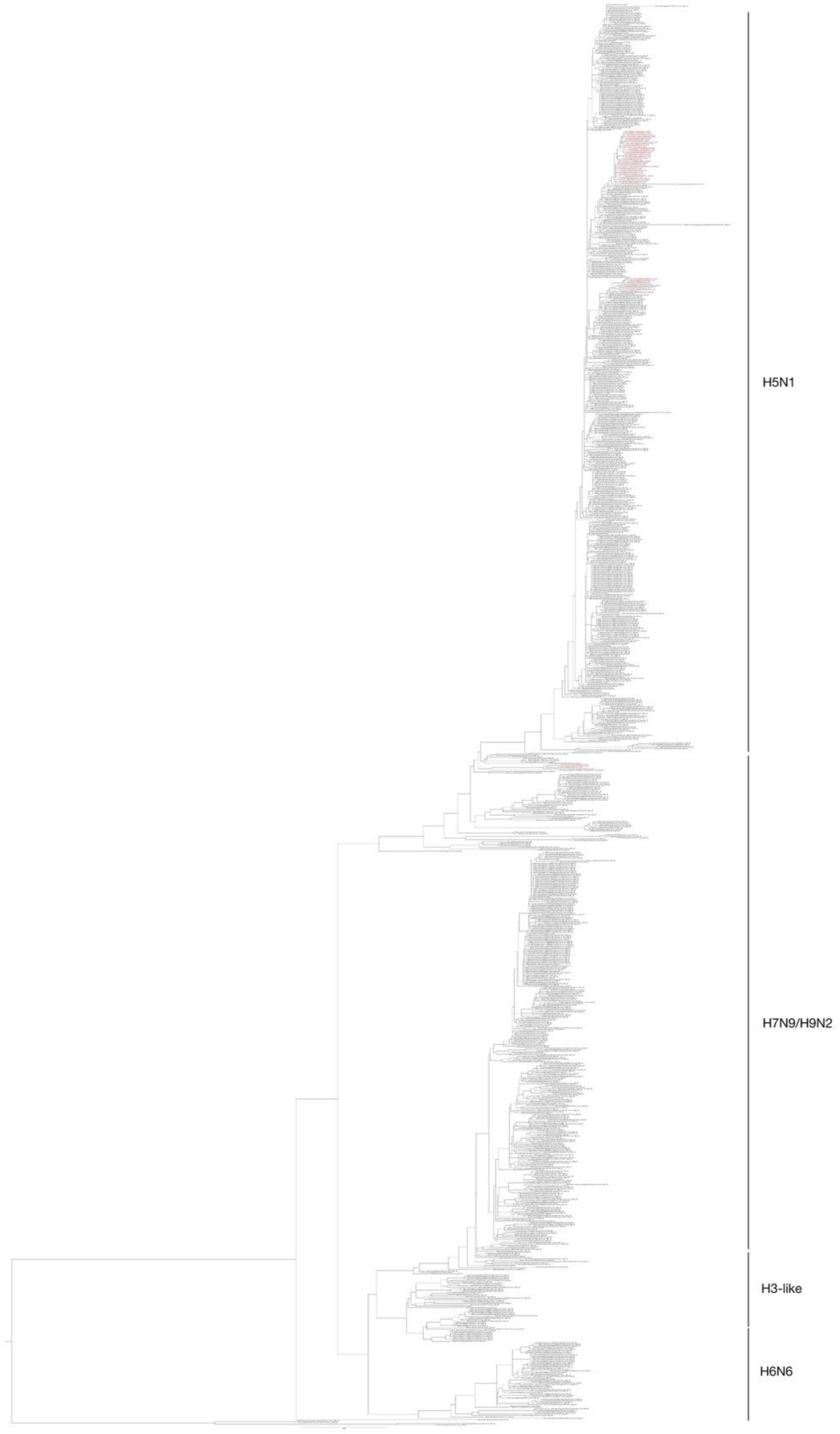

F

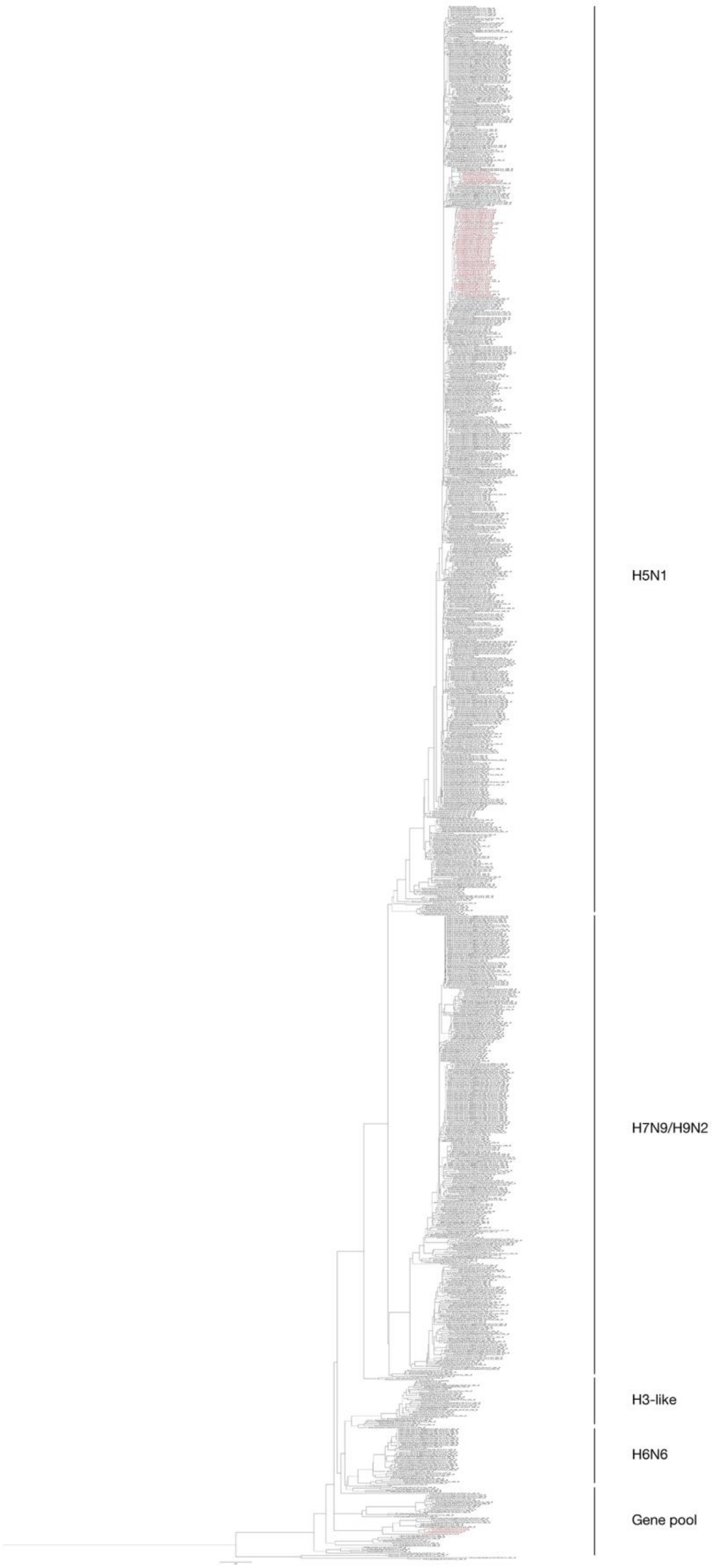

G

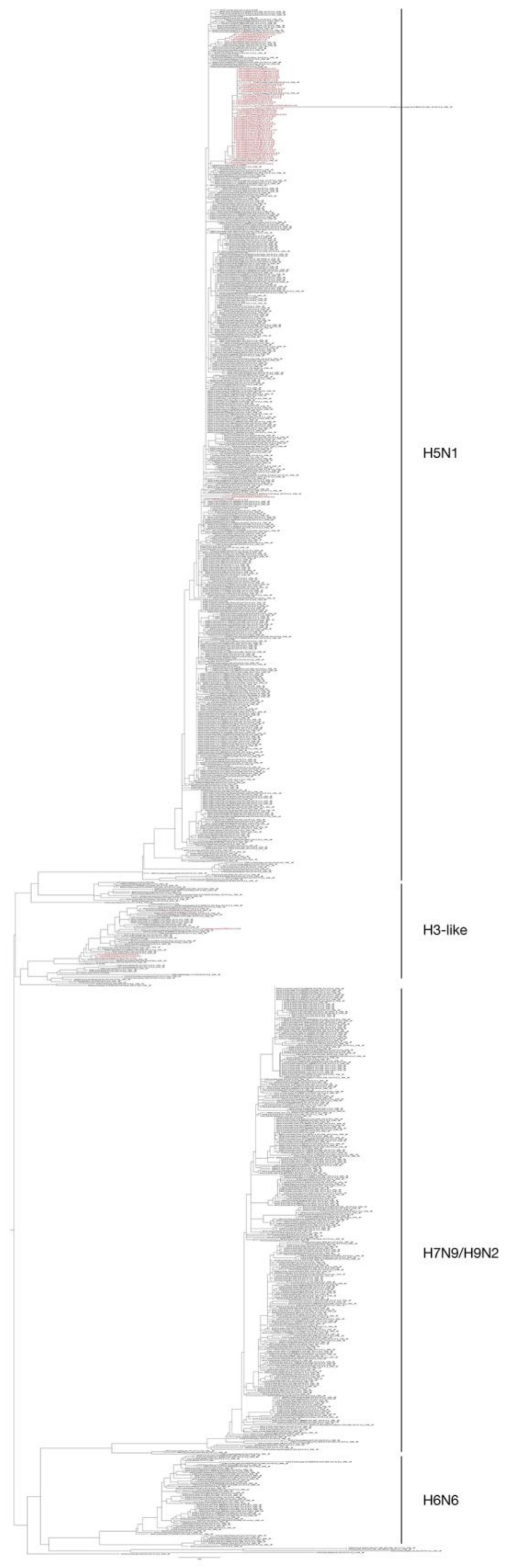

H

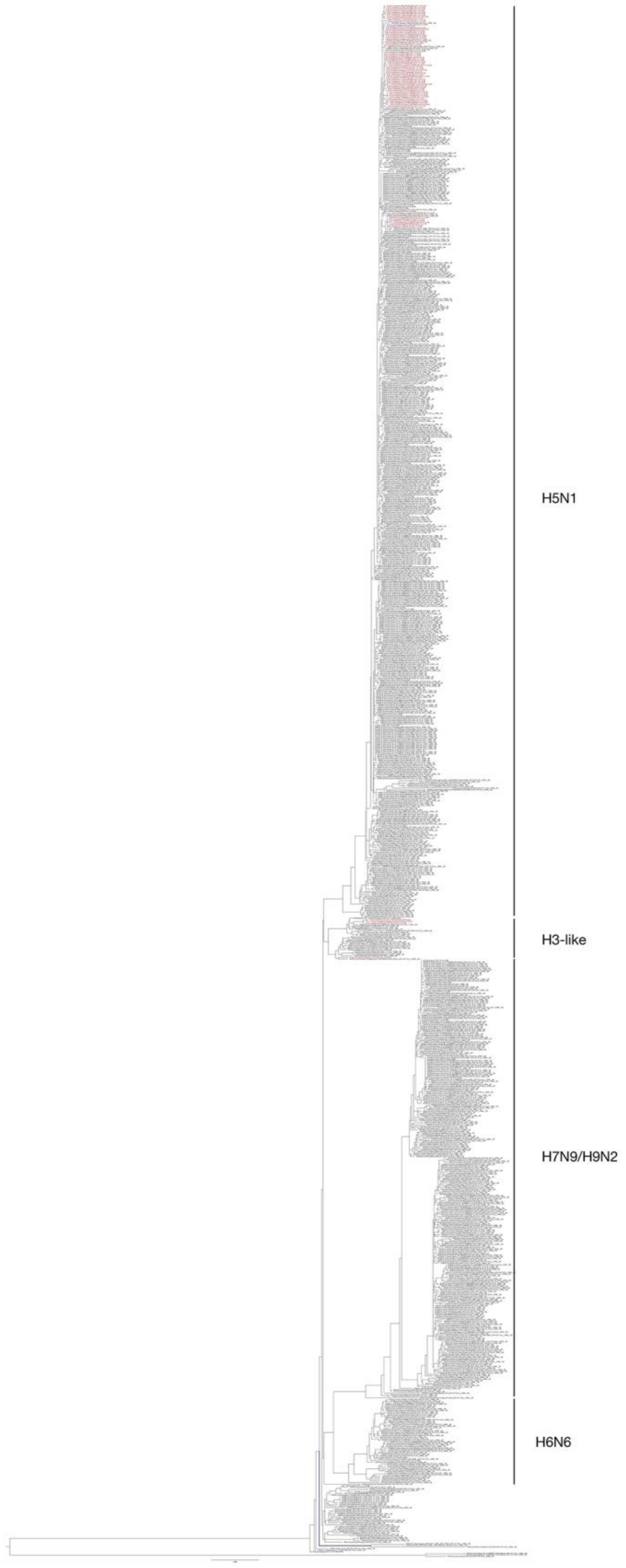

Appendix 1 Figure. Maximum-likelihood phylogenetic trees for the HA, NA, PB2, PB1, PA, NP, M, and NS genes of A(H5N6) avian influenza viruses. The viruses from the environment in this study are highlighted in red; the human case is highlighted in blue. HA, hemagglutinin; M, matrix; NA, neuraminidase; NP, nucleoprotein; NS, nonstructural; PA, polymerase acidic; PB, polymerase basic.
